# Mutations in *TP53* and *CTNNB1* in Relation to Hepatitis B and C Infections in Hepatocellular Carcinomas from Thailand

**DOI:** 10.1155/2011/697162

**Published:** 2011-06-30

**Authors:** Olivier Galy, Isabelle Chemin, Emilie Le Roux, Stéphanie Villar, Florence Le Calvez-Kelm, Myriam Lereau, Doriane Gouas, Beatriz Vieco, Iris Suarez, Maria-Cristina Navas, Michèle Chevallier, Helene Norder, Petcharin Srivatanakul, Anant Karalak, Suleeporn Sangrajrang, Christian Trépo, Pierre Hainaut

**Affiliations:** ^1^INSERM U1052, 151 Cours Albert Thomas, 69003 Lyon, France; ^2^International Agency for Research on Cancer, 150 Cours Albert Thomas, Cedex 08, 69372 Lyon, France; ^3^Departamento de Patología, Universidad de Antioquia, SIU, Carrera 51 no. 61-30, Medellín, Colombia; ^4^Grupo de Gastrohepatología, Universidad de Antioquia, SIU, Carrera 51 no. 61-30, Medellín, Colombia; ^5^Biomnis, Anatomie et Cytologie Pathologiques, 17/19 Avenue Tony Garnier, 69007 Lyon, France; ^6^Department of Virology, Swedish Institute for Infectious Disease Control (Smittskyddsinstitutet, SMI), Solna, Sweden; ^7^Cancer Control Unit, National Cancer Institute, 268/1 Rama VI Road, Bangkok 10400, Thailand; ^8^Research Division, National Cancer Institute, Rama VI Road, Bangkok 10400, Thailand

## Abstract

Hepatocellular carcinoma (HCC) may develop according to two major pathways, one involving HBV infection and *TP53* mutation and the other characterized by HCV infection and *CTNNB1* mutation. We have investigated HBV/HCV infections and *TP53*/*CTNNB1* mutations in 26 HCC patients from Thailand. HBV DNA (genotype B or C) was detected in 19 (73%) of the cases, including 5 occult infections and 3 coinfections with HCV. *TP53* and *CTNNB1* mutations were not mutually exclusive, and most of *TP53* mutations were *R249S*, suggesting a significant impact of aflatoxin-induced mutagenesis in HCC development.

## 1. Introduction

With over 600 000 new cases per year, hepatocellular carcinoma (HCC) is the 5th most common cancer and the 3rd cause of cancer mortality worldwide. Over 80% of the cases occur in non-Western countries, in particular in South-Eastern Asia [1]. The main risk factors are chronic infections by Hepatitis B (HBV) or C Viruses (HCV), alcohol, iron overload, and dietary exposure to aflatoxin, a class of mycotoxins contaminating traditional foodstuff in tropical countries. In South-Eastern Asia, HCC often occurs in a background of endemic HBV chronicity in conjunction with aflatoxin exposure. The latter is classified IARC Group 1 carcinogen for the liver and causes an inactivating mutation at codon 249 of the *TP53* tumor suppressor gene, inducing the substitution of an arginine by a serine (*R249S *mutation) [2–7]. 

The mechanisms by which HBV contributes to liver cancer are multiple, complex, and far from being fully understood [8]. In brief, three main effects can be distinguished. First, chronic infection induces inflammation and deregulation of the physiological balance between liver cell proliferation, differentiation and apoptosis. This disrupted state often leads to cirrhosis, a precursor of HCC. Second, early in the carcinogenic process, HBV DNA becomes integrated in the host cell genome, potentially acting as an insertional mutagen to deregulate adjacent oncogenes or tumor suppressors. Third, HBV expresses proteins such as HBx that interacts with a variety of cell components, affecting many aspects of transcription, proliferation, or survival. The contribution of each of the above mechanisms depends on the host immune response, the synergic effects of environmental factors, and the molecular characteristics of the strain of HBV involved. Eight major HBV genotypes have been identified (genotypes A to H), characterizing groups of viruses that show less than 8% sequence divergence between them. These genotypes differ by their geographic and ethnic distribution and their pathogenicity [9–11]. In South-Eastern Asia, the predominant genotypes are B and C, in contrast with, for example, genotype A in Northwest Europe and North America, genotype D in Southern Europe and the Middle East, and genotype E in West Africa. Disease severity has also been shown to be associated with mutation in the Basal Core Promoter (BCP) region of the viral genome, resulting in a double base substitution (*G1762A*/*A1764T*) [12]. 

Hepatocarcinogenesis is accompanied by genetic and epigenetic alterations at multiple loci, the most frequent of which are inactivating mutations in *TP53* (encoding the p53 protein, in 20 to 80% of the cases depending upon geographic and exposure contexts) and activating mutations in the N-terminus of *CTNNB1* (encoding the transcription factor *β*-catenin, in 10 to 30% of the cases) [13–17]. Based on the analysis of 137 cases of HCC from France and China, Laurent-Puig et al. [18] have proposed a model that distinguishes two main pathways of HCC, one characterized by chromosome instability, *TP53* mutations, Axin 1 mutations, HBV infection, poor differentiation, and poor prognosis, and the other characterized by chromosomal stability, *CTNNB1* mutations, absence of HBV, and tendency to form large tumors. However, it is not known whether this general model also applies to HCC from other geographic areas [14, 15, 18–22].

With incidences (Age-Standardized Rates) of liver and bile duct cancers of 33.4 in males and 12.3 in females, Thailand is a region with intermediate incidence of liver cancer. About 40 to 50% of histologically diagnosed cases are cholangiocarcinomas, which is predominant in the North-East region. The main risk factors identified for HCC are chronic HBV infection and alcohol drinking, whereas the role of HCV appears modest and there is no significant association with aflatoxin exposure as determined by measuring aflatoxin-albumin adducts in the serum. Here we describe the patterns of mutations in *TP53* and *CTNNB1* and of infection by HBV and HCV in 26 cases of primary HCC from Thailand. We found HBV DNA (genotype B or C) in 19 (73%) of the cases, including 5 occult infections and 3 co-infections with HCV. Furthermore, we found that *TP53* and *CTNNB1* mutations were not mutually exclusive, and that most of *TP53* mutations were *R249S*, suggesting a significant impact of mutagenesis by aflatoxin.

## 2. Materials and Methods

### 2.1. Patients and Tissues

Patients were recruited and specimens were obtained in the context of a study on the etiology of HCC that took place at the National Cancer Institute in Bangkok from 1987 to 1995. The basis for HCC diagnosis was an algorithm including clinical features, biochemistry (Alpha-fetoprotein levels) and imaging (ultrasonography). When confirmed by histopathology, this algorithm was found to be 95% specific for the detection of HCC. Of all cases of liver cancer detected during this period, 26 were surgically removed, snap-frozen, and bio-banked after patient's informed consent. The case series consisted of surgical resection pairs of tumor tissue (T) and nontumor tissue (NT) (the latter were available for 22/26 patients). Snap-frozen samples were stored at −80°C, transferred to IARC and analyzed according to a protocol approved by IARC Institutional Review Board. Histopathological staging and grading was performed according to Edmondson-Steiner. The clinicopathological characteristics of the patients are summarized in Table 1 and in Supplementary Table S1 available online at doi:10.1155/2011/697162. 

### 2.2. DNA and RNA Extraction

DNA and RNA were simultaneously extracted from 10 mg of liver tissue according to TEBU Masterpure extraction kit specifications (TEBU Masterpure, TebuBio Epicentre, France). DNA/RNA was re-suspended in 40 *μ*L of TE buffer. Nucleic acids were quantified by spectrophotometry, and the quality of RNA/DNA was assessed by PCR with amplification of Aldolase B gene, which is constitutively expressed in adult liver. DNA/RNA extracts were stored at −20°C.

### 2.3. *TP53* and *CTNNB1* Mutations

*TP53* mutations were analyzed by direct, automated sequencing of PCR products of exons 4 to 11 as described in the IARC *TP53* Mutation Database (http://www-p53.iarc.fr/). *R249S* mutation was further analyzed and confirmed by nested PCR/RFLP of exon 7 as previously described [23]. *CTNNB1* mutations in exon 3 (containing common activating mutations) were detected by Denaturing High Performance Liquid Chromatography (DHPLC) and sequencing. Briefly, a PCR product was generated using primers, and 3 to 10 *μ*L of this product were injected into a preheated reverse-phase column (DNASep Column, Transgenomic) equilibrated by the ion-pairing agent TEAA 0.1 M (Triethylammonium acetate). DNA was removed from the column by a linear gradient of TEAA achieved by mixing buffer A (TEAA 0.1 M) at a constant flow rate of 0.9 mL/min, and buffer B (TEAA 0.1 M and acetonitrile 25%) with 2%/minute gradient increase. The temperature for optimum separation of heteroduplex from homoduplex was calculated *in silico* by specific software (Transgenomic, San Jose, CA, USA) so that 75% of the PCR product remains double-stranded. Primer sequences and temperature used are given in Supplementary Table S2.

### 2.4. Detection of HBV and HCV

HBV DNA detection was performed using a multiplex PCR in highly conserved regions of surface (S) and core (C) genes. Two S and C fragments of 118 bp and 145 bp, respectively, were amplified as described elsewhere [24]. PCR products were analyzed by electrophoresis on 2% agarose gel and ethidium bromide staining followed by southern blotting and hybridization with a genomic length [*α* ^32^P]-dCTP HBV probe. HCV RNA was detected by seminested RT-PCR (One step RT-PCR Kit, Qiagen, France) amplifying the 5′-UTR region of HCV as described elsewhere [24]. PCR products were analyzed by electrophoresis on 2% agarose gel and ethidium bromide staining followed by southern blotting and hybridization using an HCV [*α* ^32^P]-dCTP oligo probe. 

### 2.5. HBV Genotyping, Subtyping, and Detection of “a” Loop Variants

Analysis of S gene provides genotype information significantly matching with analysis of the entire genome [25, 26]. We developed a new seminested PCR amplifying the entire S gene. First reaction was achieved with primers S_HBV123s (5′-tcgaggattggggaccctg-3′) and S_HBV848r (5′-ggaatagccccatcttttgg-3′), round settings were 95°C (5 min); 35 cycles of 95°C (30 sec), 51°C (30 sec), 72°C (1 min); then 72°C for 10 min. Second step used 2 *μ*L of first reaction and primers S_HBV123s and S_HBV 778r (5′-gaggtataaagggactcaag-3′) with similar settings to the first round. 2 *μ*L of PCR products were purified using standard ExoSap-IT (usb, Staufen, Germany) treatment, and nucleotide sequences were determined for both strand by automated, dideoxy-sequencing (sequencer ABIPrism 3100, Perkin Elmer). HBV genotypes and subtypes were determined thanks to collaboration with the Virological Department of Swedish Institute for Infectious Disease. 

### 2.6. HBV Basal Core Promoter (BCP) Variant Detection

DNA extractions of HBV DNA positive patients including T and NT tissue were tested for HBV BCP variants. Samples with detectable HBV DNA after nested PCR amplification using manufacturer's protocol were tested by line probe assay (INNO-LiPA HBV Precore Research Version, Innogenetics NV, USA) according to the manufacturer's instructions [27]. The kit probes were designed to determine the nucleotide sequences at positions 1762 (A versus T) and 1764 (G versus A and G versus T) in the BCP region. Determination of Pre-Core (PC) mutation at HBV codon 1896 (G versus A) was achieved in the same reaction.

### 2.7. Immunohistochemistry

Tissue fragments were fixed in 10% buffered formalin and paraffin embedded according to standard protocols. Deparaffinized tissue sections were labeled using standard protocols with CM1 antibody (Ab) (rabbit polyclonal immunoglobulin G anti-human p53, 1/500, Novacastra Laboratories Ltd., Newcastle, United Kingdom) recognizing all isoforms of p53 protein. HBV- and/or HCV-positive tumor sections were labeled independently with a set of antibodies to either HBxAg (rabbit polyclonal Ab used at 5 *μ*g/mL) [28] or E2 protein for HCV positive sections (D4.12.9 monoclonal Ab at 0.2 *μ*g/mL) [29]. Fixed antibodies were detected using biotinylated immunoglobulin G, streptavidine-peroxidase, and diaminobenzidine (Vector Laboratories, Inc., Burlingame, CA).

## 3. Results

### 3.1. Patients and Tumor Characteristics

Table 1 lists the clinicopathological characteristics of patients and tumors analyzed. Age range was from 17 to 73, with a majority of cases occurring before 50 years of age (62.5%) and a male/female ratio of 5 : 1. HBsAg status was positive in 13 of 23 patients for whom this information was available (56.5%). Edmondson and Steiner's grades of tumors were equally distributed between scores ≥G3 and <G3. Most of adjacent NT tissues showed an activity score <2 and a fibrosis score ≥2 in the METAVIR scoring system. Most HCC were mainly trabecular (69.3%). Pleiomorphic or pseudoglandular types represented 11.5% and 7.7%, respectively. In 3 cases, high levels of necrosis or tissue degradation were observed precluding the precise assessment of HCC morphology. Cirrhosis was diagnosed in 7 of the 20 NT tissues.

### 3.2. Mutations in *TP53* and *CTNNB1* Genes

**Figure 1:**
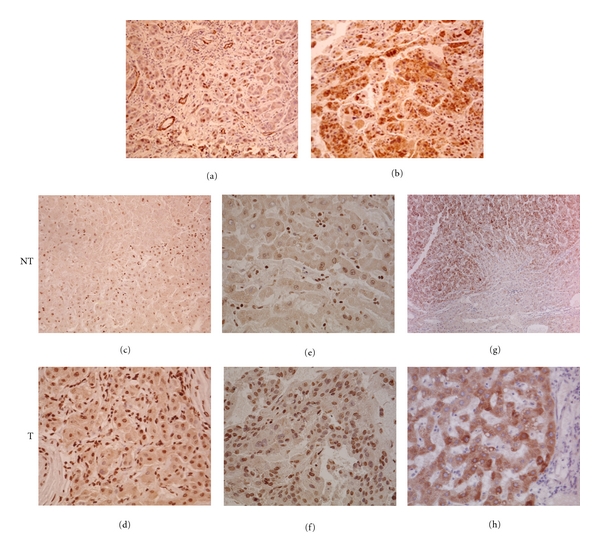
Immunostaining of HCC sections ((a)–(g) × 200, (h) × 400). Accumulation of p53 in HCC with *R249S* (a) and *P278R* (b). Accumulation of HBxAg in hepatocytes of nontumoral (NT) and tumoral (T) tissues of HCC with overt HBV infection (c,d) or occult HBV infection (e,f). Accumulation of HCV E2 protein in tumor section of overt HBV/HCV (g) or occult HBV/HCV (h) coinfected HCC.

*TP53* mutations were detected in 9 cases (34.6%), with a high proportion of *R249S* (present in 7 cases, 77.8%). This mutation was associated with a low level of p53 nuclear accumulation in the tumor (<10% of cells stained, 6 cases) or with retention of p53 in the cytoplasm (1 case) (Figure 1(a)). Immunostaining of *R249S*-positive sections with an antibody to aflatoxin-DNA adducts was negative (data not shown). Two other mutations were at codon 278 (CCC to CTC, *P278R*) and codon 331 (CAG to CAT, *G331H*). *P278R* falls within the DNA-binding domain and the mutant protein shows loss of transactivation activity towards 8 different p53-dependent promoters in yeast functional assays [30]. This mutation was associated with accumulation of the protein in 10–20% of the cells (Figure 1(b)). In contrast, *R331H* falls in exon 9 next to the p53 oligomerization domain and does not appear to significantly suppress p53 transactivating capacity in a yeast functional assay [30]. This mutation does not result in p53 accumulation (data not shown). Three specimens exhibited p53 accumulation despite the presence of wild-type (WT) *TP53 *sequences (data not shown).

Six mutations in exon 3 of *CTNNB1 *(23%) were found by DHPLC and direct sequencing. Mutations were exclusively found in tumors at codons specifying serines or threonines in the N-terminus. These phosphorylation sites are part of the GSK3-*β* box. Four out of the six tumors with *CTNNB1* mutation also had a *TP53* mutation (three *R249S* and one *P278R*) (Table 2).

### 3.3. Molecular Patterns of Hepatitis Viral Infections

**Figure 2:**
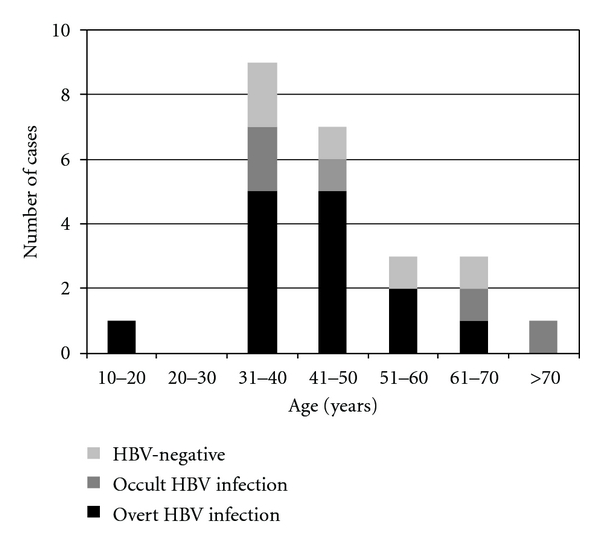
Age distribution and HBV infection status of HCC cases.

HBV DNA was detected in both T and NT specimens of 19 HCC patients (73%), whereas 4 HCC cases (15.4%) were positive for HCV RNA, 3 out of 4 being coinfected with HBV. When comparing HBV DNA with HBsAg status, 11 cases were positive for both, 5 cases were positive for DNA only (occult infection), and 2 cases were positive for HBsAg only (note that nontumor tissue was not available for these two cases). Thus, of the 10 HBsAg negative cases, 5 harbored HBV DNA, indicating a proportion of 50% of occult infections among HBsAg negative patients. Only one of these 5 patients with occult HBV infection was positive for HCV. Occult infection was detected in all age groups, but was more frequent in patients over 51 (3/5, 60%) than under 50 (3/14, 21.4%) (Figure 2).

Preamplification of Pre-C/C region prior to INNO-LIPA reverse hybridization was successfully performed in 9 out of 19 HBV DNA-positive specimens. The BCP double variant A1762/T1764 was found in 7 cases (77.8%). Distribution of BCP double variant and wild-type HBV populations was not restricted to tumoral tissues. The PC variant was detected in the NT tissue of one patient (data not shown, see Supplementary Table S1). 

The entire S gene was sequenced in 11/19 HBV DNA-positive specimens. The two main HBV genotypes were C and B, found, respectively, in 81.8% (9/11) and 18.2% (2/11) of patients. Several mutations were detected in the region corresponding to the “a” loop determinant (residues from 124 to 147) [31]. Two mutations have been previously described as “escape” mutations in vaccinated subjects (*G145R* and *I126N*) [32, 33]. Both were found in patients with occult HBV infection (Supplementary Figure S3). In specimen 26, we detected a double mutation *A126I*/*P127T*, which has not been reported before.

HBxAg was strongly expressed in 80% of HBV-positive cases. The pattern of HBxAg accumulation differed between T and NT tissues. In the latter, we observed cytoplasmic accumulation in 15–60% of cells, with nuclear accumulation in some hepatocytes (Figure 1(c)). In the former, accumulation was stronger in regeneration nodules and nuclear compartments were strongly stained (Figure 1(d)). The same staining pattern was observed in either overt or occult HBV infections (Figures 1(e) and 1(f)).

### 3.4. Concordance between Viral Infection, Tumor Morphology, and Mutations in *TP53* or *CTNNB1*

**Figure 3:**
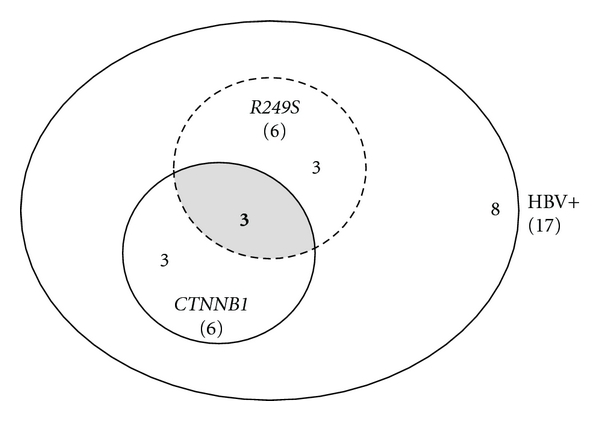
Distribution of *TP53* and *CTNNB1* mutations in overt and occult HBV-infected cases. All HBV-positive cases were counted from Table S2. Five cases with nonavailable *CTNNB1* status were excluded, giving a total number of 17 cases. Total numbers are indicated in brackets. Six cases were mutated in either *CTNNB1 *or in *TP53 *(*R249S*) and 3 of them harbored both mutations. Finally, 8 cases were wildtype for both genes or mutated at other codons in *TP53*.

Tumors were classified according to their HBV status as occult, overt, or negative HBV infection. There was no difference in relation to age, sex, tumor grade, activity/fibrosis scores, and incidence of HCV infection between the three groups (Table 3). HBV genotypes and PC/BCP mutations were equally distributed within the three groups. However, trabecular morphology was more common among HBV-infected subjects, regardless of occult or overt status. Regarding mutations, *CTNNB1* mutation rates did not differ between the three groups but *R249S *was strongly associated with overt HBV infection. Accumulation of p53 in tumors with wild-type *TP53* sequences was identified exclusively in HBV-negative tumors, one of which was HCV RNA-positive (see Supplementary Table S1). Overall, these results do not substantiate that *TP53* and *CTNNB1* mutations fall in distinct subtypes of HCC (Figure 3). However, they confirm the strong concordance between *R249S* and overt chronic HBV infection.

## 4. Discussion

Most of the current knowledge on the mechanisms of HCC pathogenesis is based on studies developed in Western Europe or in the US. However, these two regions hold less than 25% of the world annual cases of HCC. Given the strong geographic differences in the distribution of risk factors, studies in other areas are mandatory to obtain a more precise picture of the complexity of interactions between mutations and viral infections. In particular, countries such as Thailand represent interesting areas because of the coexistence of multiple viral, lifestyles, dietary, and environmental risk factors. While detailed assessment of these factors is beyond the scope of this work and will require extensive case-control comparison studies, the present pilot study shows notable differences when compared with the results of studies in Western countries. These differences are (1) the high prevalence of *R249S*, which is unexpected in a country where exposure to aflatoxin through the diet is considered as relatively low; (2) the high prevalence of HBV infections (80.8%), taking into account occult infections; (3) the relatively modest role of HCV infection, which is present in only a small proportion of the cases, mostly in conjunction with HBV infection; (4) the coexistence of *TP53* and *CTNNB1* mutations in a proportion of the cases (3 cases among the 11 with either *TP53* or *CTNNB1* mutations), inconsistent with a systematic mutual exclusion between two distinct pathways of carcinogenesis.

There is evidence that *R249S* is caused by direct adduction of aflatoxin metabolites on the third base of codon 249. This mutation is very specific for HCC in high incidence areas of China or sub-Saharan Africa, and there is a good overall concordance between the prevalence of this mutation in HCC and the levels of exposure to aflatoxins in the general population. In Thailand, the population exposure is considered as low to intermediate. In 1993 a study conducted on a series of 15 HCC cases from central Thailand reported only one *R249S *(6.7%) [3]. However, more recently Kuang and collaborators [4] reported a prevalence of *R249S* of 24% in HCC cases from Chiang Mai in the Northern part of Thailand. Our results (26.9%) are compatible with their results [4] and suggest that exposure to aflatoxin in the general population of Thailand may be higher than previously recognized, despite absence of detectable aflatoxin-DNA adducts in liver tissues of patients. In line with this notion, a recent assessment of aflatoxin contamination in Thailand in two basic dietary components, corn and peanuts, has shown mean aflatoxin levels which are 3 to 5 times higher than the Thailand Regulation limit [34]. Although these levels are about 4 times lower than those reported in populations where HCC is highly prevalent, they suggest that aflatoxin may still play an important role as a cofactor with chronic HBV carriage in causing HCC in Thailand.

Patients with *R249S* were all HBV carriers, and they tended to be younger than those without mutation or with mutation at other codons. This pattern is similar to the one of HCC in areas of high HBV endemicity combined with high exposure to aflatoxin [23]. Interestingly, we noted that occult HBV infection represented 27.8% of all HBV-positive cases, compatible with previous studies in China (26%, *n* = 132) or Taiwan (29%, *n* = 31) [35, 36]. None of these cases harbored an *R249S* mutation. These observations are consistent with the hypothesis that occult HBV infection may play a causal role in the pathogenesis of HCC [37, 38].

In agreement with previous studies [39], HBV genotype C was predominant (81.8%), followed by genotype B (18.2%). The BCP double-mutation 1762T/1764A was detected in all of the 6 patients analyzed, either in the tumor (1 case), the adjacent tissue (2 cases), or both (3 cases). Thus, despite published results suggesting that this mutation may be associated with increased severity of infection and cirrhosis, our results do not suggest selection of the mutation during tumor progression.

Occult hepatitis may result from different mechanisms including defective HBsAg expression (due to, e.g., structural or regulatory mutations in S gene [24, 40]) or inhibition of HBV replication due to HCV co-infection [41, 42]. Of the S gene sequences analyzed in the present study, two exhibited escape mutations in the antigenic determinant “a” (G145R + I126N for THAI_10_ and G145R for THAI_27_); while we did not detect any nucleotide change in the S sequence of the 7 cases with overt HBV infection. A third sequence (THAI_26_) exhibited a double nucleotide substitution *I126A* + *P127T* that has not been reported so far. The consequences of this mutation are unknown, although the contiguous isoleucine to proline substitution may induce structural changes explaining the absence of HBsAg response.

In our study, HCV infection rate is 5.25-fold lower than HBV, confirming a low to intermediate contribution of HCV in HCC development. However, it is important to note that a strong accumulation of HCV envelope protein was detected in regeneration nodules of either mono- or coinfected samples (Figures 3(g) and 3(h)), suggesting active replication of the virus within tumor cells.

Studies on genetic alterations in HCC identifying two distinct pathways for hepatocarcinogenesis lead to consider that mutations in *TP53* and *CTNNB1* rarely occur simultaneously in liver tumors. However, this model is essentially based on results obtained on tumors from Western countries and it is unclear whether it also applies to HCC in a context of aflatoxin exposure. Our results suggest that *CTNNB1* mutations may be present in HBV-infected tumors together with *R249S*. It should be considered that, in the context of aflatoxin exposure and chronic HBV infection, *R249S* is likely to occur as an early event, which may contribute to genetic and chromosomal instability and thus facilitate cell proliferation and survival in the hostile conditions that result of the combined exposure. This, in turn, may increase the risk of acquisition of mutations in many other genes including *CTNNB1*. In contrast, in a context of low aflatoxin exposure, events other than *TP53* mutations are likely to take place as early steps. *CTNNB1* mutations may represent such an early event, which may be conditional for the entire sequence of subsequent events during tumor progression. In such a scenario, *TP53* mutations may occur as a late event in a subset of cancers. Thus, we propose that HCC that arises in a context of aflatoxin exposure may develop according to a specific sequence of genetic events that may include both *TP53* and *CTNNB1* mutations. The identification of key steps in this sequence will require extensive comparative studies on gene alteration and expression patterns from HCC from different geographic areas and etiological contexts.

## Figures and Tables

**Table 1:** Clinicopathological data of HCC cases.

	Frequency (%)
Age (*n* = 24)	
≥50	9 (37.5)
<50	15 (62.5)
Mean age ± SD	46 ± 13

Gender (*n* = 24)	
male	20 (83.3)
female	4 (16.7)

HBsAg status (*n* = 23)	
Positive	13 (56.5)
Negative	10 (43.5)

Edmondson and Steiner's grade of T tissues (*n* = 25)	
≥G3	12 (48.0)
<G3	13 (52.0)

METAVIR Score of NT tissues (*n* = 20)	
Activity < 2	16 (80)
Activity ≥ 2	4 (20)
Fibrosis < 2	5 (25)
Fibrosis ≥ 2	15 (75)

HCC morphology (*n* = 26)	
Trabecular type	18 (69.3)
Pleiomorphic type	3 (11.5)
Pseudo-glandular type	2 (7.7)
Necrotic tissue	3 (11.5)

T = tumor, NT = nontumoral tissue.

**Table 2:** Distribution of HBV/HCV infection, *CTNNB1* mutations and clinicopathological data according to *TP53* mutation status.

	MUT *TP53*		WT *TP53*	Total
	*R249S*	Other		
*TP5*3	7	2	17	26
*CTNNB1*	3	1	2	6
HBsAg positive	5	1	7	13
HBV DNA	6	2	11	19
HCV	1	1	2	4
No HBV nor HCV	0	0	4	4

Tumor grade				
≥3	5	2	6	25*
<3	2	0	10	
Cirrhotic NT tissue	3	2	2	7

Age	37.6 ± 12.8	53	48.2 ± 12.41	
Sex				
M	5	2	13	24^†^
F	0	0	4	

MUT = mutant, WT = wildtype, NT = nontumoral tissue, *grade is missing for one WT *TP53* case, ^†^age and sex data are missing for two *R249S* cases.

**Table 3:** Distribution of *TP53*/*CTNNB1* mutations and clinicopathological data according to HBV infection status.

	Overt HBV infections *n* = 13*	Occult HBV infections *n* = 5	Negative for both HBsAg and HBV DNA *n* = 5	Total
*TP53*				
*R249S*	5	0	0	5
Other mutation	1	1	0	2
WT	7	4	5	16

*CTNNB1*				
MUT	3	2	0	5
WT	7	3	4	14

HCV	2	1	1	4

Sex				
M	13	4	3	20
F	0	1	2	3

Grade				
≥3	7	2	2	11
<3	6	3	3	12

HCC morphology				
Trabecular	11	5	2	18
Pleiomorphic	2	0	1	3
Pseudoglandular	1	0	1	2
Necrosis	1	0	2	3

Age	43.2 ± 12.12	51.4 ± 15.2	49.4 ± 13.7	

WT = wildtype, MUT = mutant, *three HBV-DNA positive cases with missing data on serology were omitted in this table, ^†^ *CTNNB1* was not amplifiable in four cases, three cases with overt HBV infection and one HBV-negative case.
